# Antibiotic Therapy in Dentistry

**DOI:** 10.1155/2021/6667624

**Published:** 2021-01-28

**Authors:** Hanie Ahmadi, Alireza Ebrahimi, Fatemeh Ahmadi

**Affiliations:** ^1^Student Research Committee, School of Dentistry, Shiraz University of Medical Sciences, Shiraz, Iran; ^2^Student Research Committee, Shiraz University of Medical Sciences, Shiraz, Iran; ^3^School of Pharmacy, Shiraz University of Medical Sciences, Shiraz, Iran

## Abstract

Dental caries, pulpal necrosis, trauma, and periodontal diseases can result in dental infections which could have severe consequences that affect both soft and hard tissues of the oral cavity. Dental infections commonly present with symptoms of pain, fever, and swelling. Surgical and endodontic treatments are the early management of infected teeth, followed by antibiotic therapy. Some alternative methods also exist for treating infection such as low-level laser therapy and photodynamic therapy. Antibiotics are generally used in dental procedures to treat odontogenic infections, nonodontogenic infections, local infection, focal infection, and prophylaxis. Antibiotic prophylaxis is prescribed for patients with immunosuppressed conditions, infective endocarditis, metabolic disorders, and patients with prosthetic joints. To reduce the complications of unnecessary antibiotic prescriptions especially bacterial resistance, comprehensive guidelines should be established. It has been noted that only about 12% of dentists adequately and correctly prescribe antibiotics, which shows the importance of comprehensive guidelines. Antibiotics prescription may result in some adverse effects such as hypersensitivity reactions and dermatological and allergic disorders. Furthermore, unnecessary prescription of antibiotics could result in several serious sequelae, for example, bacterial resistance, gastric and hematological problems, and diversion of bacterial microbiota. The present review attempts to summarize the indications of antibiotic therapy in dentistry and discuss the common types of antibiotics that are routinely used in dental practice based on pharmacologic classes. Moreover, types of antibiotics that are considered safe during pregnancy and childhood are also reviewed.

## 1. Background

Orofacial infections are commonly categorized as odontogenic and nonodontogenic. The conditions that originate within a tooth and dental supporting structures are called odontogenic infections. On the other hand, teeth structures are not involved in nonodontogenic infections [1, 2]. Dental caries, pulpal necrosis, dental trauma, and periodontal diseases can result in dental infections which could have severe consequences that affect both soft and hard tissues of the oral cavity. According to a previous study, Gram-positive cocci are responsible for about 65% of orofacial infections, and Gram-negative bacilli could be found in 25% of patients' oral specimens [3]. Orofacial infections mostly occur during the age of 21–40; besides, the prevalence of the disease is not gender-related [1, 4].

Dental infections are commonly presented by symptoms of pain and swelling in the oral area. These infections should be treated as soon as possible, as they may lead to severe and irrecoverable consequences such as osteomyelitis, brain abscess, airway obstruction, carotid infection, sinusitis, septicemia, meningitis, cavernous sinus thrombosis, orbital abscess, and loss of vision [5]. It has been noted that the most common prevalent feature of orofacial infections are dentoalveolar abscess [3].

Dental infections could be cured by surgical interventions, endodontics therapy, and antibiotic prescriptions [4]. The early surgical management of the infected tooth should be carried out to prevent further consequences; this may include debridement, irrigation, and incision and drainage (I&D) in severe cases [6]. Furthermore, in patients with the signs of systemic involvement, administration of intravenous antibiotics according to bacterial cultures and sensitivity is suggested [5, 7]. Present guidelines indicate that antibiotics should be prescribed after the elimination of the infectious sources. These should be prescribed for 2-3 consecutive days after surgical treatments. Longer durations of antibiotic therapy were not found to be significantly beneficial and are not recommended [8]. This could result in the unnecessary prescription and a longer duration of antibiotic therapy that may have serious consequences [9].

Previous studies demonstrated that about 12% of dentists adequately prescribe antibiotics as a prophylactic intervention and treatment [7]. In this regard, previous reports have mentioned that the most common antibiotic that is prescribed in dental practice is amoxicillin followed by amoxicillin and clavulanic acid (Table 1) [10]. Antibiotics prescription may result in some adverse effects such as hypersensitivity reactions and dermatological and allergic disorders [11]. Furthermore, unnecessary prescription of antibiotics could result in several serious complaints, for example, bacterial resistance, gastric and hematological problems, and diversion of bacterial microbiota [12, 13]. Besides, this could lead to oral bacterial resistance which is considered a growing concern in dentistry and medicine as well. To prevent these problems, antibiotics should be prescribed in a narrow spectrum and be limited to acute infections. Moreover, further education and investigation should be conducted to prevent and reduce the problem of antibiotic resistance [14].

Since a few decades ago, the rate of odontogenic infections has tended to be decreasing. This could be because of the rising education of individuals about oral hygiene and the development of oral healthcare products [4]. Conversely to the abovementioned fact, the orofacial infections are still considered as a worldwide problem. The lack of sufficient strategies for antibiotic prescription in dentistry has been a concern for many dental practitioners, and more education is needed in this regard [14]. To decrease the prevalence of dental infection and its consequences, comprehensive guidelines are needed for the treatment of the condition.

**Figure 1:**
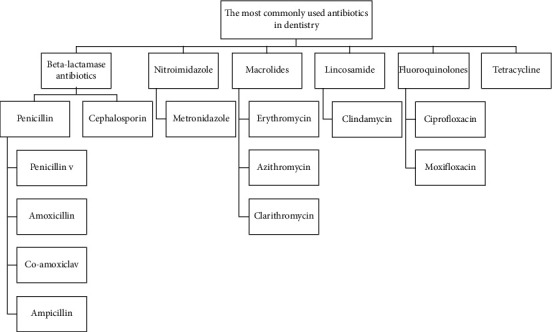
The most commonly used antibiotics in dentistry.

In this review, we aim to provide some applicable data for dentists to prescribe appropriate types of antibiotics. At first, common types of antibiotics that are considered safe during pregnancy and childhood are mentioned. Then, the common cases that need antibiotic therapy or prophylaxis and the most useful and commonly used antibiotics in dentistry are reviewed (Figure 1).

## 2. Indications of Antibiotics

**Figure 2:**
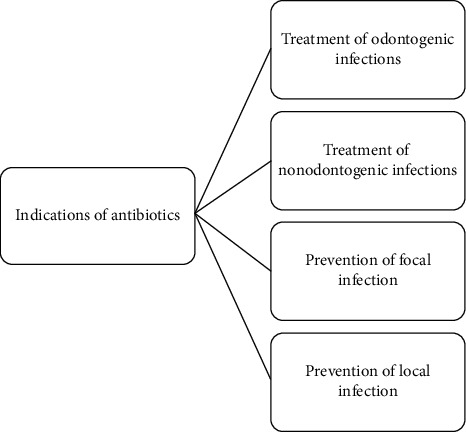
Indications of antibiotics in dental practice.

Antibiotics are suggested in the cases of prophylaxis for local and focal infections, besides, for the treatment of odontogenic and nonodontogenic infections [13, 15]. Antibiotics are not indicated for all odontogenic infections; they should not be used instead of removal of the source of infections [16]. In the case of infection, I&D, debridement, and endodontic management followed by systemic antibiotic therapy are recommended [17]. Moreover, the practitioners should also bear in mind that the antibiotic prophylaxis is indicated in a few specified conditions [18].  Figure 2 summarizes the indications of antibiotics in dental practice.

Antibiotic prophylaxis is a necessary option in cases of immunosuppressed patients, patients with a history of cancer, individuals with infective endocarditis, patients with metabolic disorders (such as diabetes and splenectomies), patients with prosthetic joints, in-dwelling catheters, neurosurgical shunts, valvular heart diseases, surgical pulmonary shunts, hypertrophic cardiomyopathy, mitral valve prolapsed, and prosthetic heart valves [19, 20]. In susceptible patients, some procedures enhance the risk of infection such as dental extraction, surgical periodontal procedures, dental implant placement, reimplantation of teeth, endodontic procedures or endodontics surgeries, subgingival placement of antibiotic fibers or strips, and intraligamentary local anesthetic injections [19]. Prophylaxis for healthy patients is also suggested in special dental practices, such as surgery for benign tumors, bone grafting, implant placement, periapical surgery, and removal of impacted teeth [20].

Antibiotic prescription is recommended in acute infection conditions such as necrotizing ulcerative gingivitis, stage III-grade C/incisor-molar pattern periodontitis (formerly referred to as localized aggressive periodontitis), acute periapical abscess, cellulitis, local or systemic spreading of infection in the periodontal abscess, pericoronitis, periimplantitis, infection of deep fascial layers of the head and neck, and in the case of fever and/or malaise [6, 20, 21].

## 3. Antibiotic Use in Pediatric Dentistry

Anatomical and physiological differences between children and adults such as the amount of their body water and fat, the maturation of the immune system, the volume of protein, and the level of liver enzymes should be considered while prescribing antibiotics for children [22]. Dentists treat children with antibiotics to reduce the risk of bacteremia caused by dental infections; however, antibiotic therapy should not be used as an alternative method for elimination of an infection source [23]. Furthermore, antibiotic resistance owing to inappropriate use, prescribing antibiotics in the wrong situation and for a too long period in children is a global concern [24]. Therefore, dental practitioners should be aware of proper antibiotic choices and indications of antibiotic therapy for children under 13 years [24]. Common types and forms of antimicrobial agents used in pediatric dentistry are listed in Tables 2 and 3.

## 4. Antibiotic Therapy during Pregnancy

The physiological changes of pregnancy can affect the condition of the oral cavity such as increasing the risk of gingivitis and pyogenic granuloma [25]. Preventive or therapeutic interventions during this period should be carried out to preserve the health of both mother and her neonate, enhance maternal oral health, and reduce children's future oral problems [26]. In this regard, it has been mentioned that the mothers with poor oral hygiene who have a higher number of microorganisms in their saliva, especially *Streptococcus mutans*, can easily transmit the infection to the infant causing several serious problems for them [26]. It should be also noted that most of the dental procedures are not emergencies and can be postponed after delivery; however, acute dental infections should be managed during pregnancy [25–27].

The drug prescription during the pregnancy should be done more cautiously, as the inappropriate prescription could irrecoverably harm the fetus. In dental practice, the main agents that are commonly used during pregnancy and are considered to be safe during this period are analgesics, anesthetic agents, and antibiotics [26]. Food and Drug Administration (FDA) has classified drugs into 5 groups (A, B, C, D, and X) based on their risk factors during pregnancy (Table 4), and most of the antibiotics are classified to be in class B of FDA arrangement [28]. Furthermore, the pregnant patients should receive a complete adult dose with the usual length of treatment [27].

## 5. The Most Common Prescribed Antibiotics

### 5.1. Beta-Lactams

Beta-lactam antibiotics are the antimicrobial agents that contain beta-lactam ring in their molecular structure (this ring includes a three-carbon and one-nitrogen cyclic amine structure) [7, 29]. This group of antibiotics is bactericidal agents that act against many Gram-positive, Gram-negative, and anaerobic bacteria via inhibiting the synthesis of the cell wall [7]. Beta-lactam antibiotics are categorized into five classes: penicillin, cephalosporins, penems, carbapenems, and monobactams [30].

The overuse and misuse of penicillin and cephalosporins has resulted in an increased rate of bacterial resistance, caused by the production of beta-lactamase. Moreover, the risk of resistance might be increased if penicillin is administered simultaneously with other antibiotics, for instance, metronidazole [31]. Allergic reactions caused by the release of immunoglobulin E (IgE) mediators are among the common side effects of beta-lactams and might include rashes, pruritis, and even anaphylactic shock [29].

### 5.2. Penicillin

Penicillin is a narrow-spectrum antibiotic that was discovered from a rare variant of *Penicillium notatum* [32]. The most common types of penicillin that are being administered for treatment of odontogenic infections are penicillin V, amoxicillin, and amoxicillin/clavulanic acid, and studies show that they have almost the same efficacy regarding the treatment of dental infections [21]. According to previous investigations, nearly 70% of bacteria isolated from odontogenic infections were susceptible to penicillin [33]. Commonly, penicillin is considered to be the first-line drug and the gold standard for the treatment of odontogenic infections because of its cost-effectiveness, low incidence of side effects, and appropriate antimicrobial activity [21, 34]. Despite these benefits, the drug might cause various side effects in certain patients, including rash, nausea, gastric irritation, diarrhea, and hypersensitivity reactions such as skin reactions [13]. It has been mentioned that about 10% of people might present some levels of hypersensitivity to the drug; however, 90% of them can tolerate penicillin [34]. Should the patients have a history of hypersensitivity to the drug or a positive skin test, clindamycin could be administered instead of penicillin [21].

#### 5.2.1. Penicillin V

Compared with penicillin G, penicillin V stays for a longer time in blood circulation [12]. Tablet of 500 milligrams (mg) penicillin V is recommended every 6 hours taking by mouth [17]. Moreover, 2–4 g penicillin V every 4–6 hours combined with 500 mg metronidazole intravenous (IV) or orally every 8 hours could also be prescribed [31].

#### 5.2.2. Amoxicillin

Amoxicillin is a penicillin antibiotic that acts against Gram-negative bacilli [6, 35]. Amoxicillin is commonly considered to be the first line of treatment in nonallergic patients [36]. It is the most frequently prescribed antibiotic accounting [37]. Some practitioners also prefer to administer the combination of amoxicillin and metronidazole or amoxicillin/clavulanate to treat odontogenic infection [38, 39]. The therapeutic dosage for amoxicillin is 500 mg every 8 hours or 1000 mg every 12 hours [21].

#### 5.2.3. Amoxicillin with Clavulanic Acid (Co-Amoxiclav)

Amoxicillin with clavulanic acid (co-amoxiclav) is a broad-spectrum antibiotic that is believed to be the second most prescribed antibiotic by dentists [9]. It has been shown that all the bacteria that were extracted from an odontogenic abscess were susceptible to the agent [33]. Besides, in the case of amoxicillin resistance, the administration of co-amoxiclav or metronidazole is suggested [40]. A high dose of co-amoxiclav (875/125 mg every 8 hours or 2000/125 mg every 12 hours) is a proper choice in the cases of severe odontogenic infections, such as abscess and pulpitis [41]. The dental practitioners should be aware that the drug could result in some levels of hepatotoxicity; besides, it can change the orogastrointestinal normal microbiota causing candidiasis or even *Clostridium difficile* infection [42].

#### 5.2.4. Ampicillin

Ampicillin is categorized as a broad-spectrum beta-lactam antibiotic that has bactericidal activity [7]. The drug antibacterial activity mostly covers the Gram-positive bacilli, but it acts less effectively than amoxicillin [6]. Moreover, ampicillin mainly acts against aerobic bacteria, and it could be simultaneously prescribed with metronidazole to more efficiently fight anaerobic bacteria of odontogenic infections [43]. The agent is commonly used for patients who cannot orally take drugs, and the prophylaxis dosage is 2 mg IV or intramuscular (IM) half an hour before the procedure [20]. Furthermore, ampicillin-sulbactam could be prescribed 3 g intravenously every 6 hours [29]. The coadministration of ampicillin and clindamycin could increase the risk of pseudomembranous [44].

#### 5.2.5. Cephalosporin

Cephalosporins are classified in beta-lactam antibiotics and can inhibit the biosynthesis of bacterial cell walls [38]. Cephalosporins can act against aerobic bacteria, and their combination with metronidazole could cover both aerobic and anaerobic bacteria [43]. Cephalexin and cefazolin are among the most commonly prescribed first-generation cephalosporins in dental practice [45]. Cephalexin could be prescribed for penicillin-allergic patients, with the dosage of 2 g orally 1 h before dental procedures [20]. Cefazolin is suggested for patients who are allergic to penicillin and cannot take the medication by mouth, with the dosage of 1 g IV or IM 30 minutes before the procedure [20]. Older studies recommended not to use cephalosporins in penicillin-allergic patients, while more recent investigations showed that there is little cross-activity between penicillin and cephalosporins [46]. Studies also mentioned that while the cephalosporins have few side effects and better antimicrobial activity, amoxicillin is still the drug of choice for the treatment of odontogenic infections [46, 47]. The patients who were treated with cephalosporins might have higher risks of colonization of *Candida albicans* and yeast [48].

### 5.3. Nitroimidazoles

Nitroimidazoles are commonly administered to treat parasitic and anaerobic bacterial infections. Nitroimidazoles include metronidazole, nimorazole, and tinidazole [49, 50]. It has been noticed that dental practitioners tend to prescribe metronidazole for the treatment of acute infections, as it has great antianaerobic bacterial activity and low risk of toxicity [49, 50].

#### 5.3.1. Metronidazole

Metronidazole has bactericidal activity and acts against anaerobic microorganisms by inhibiting the nucleic acid synthesis; the agent also showed antiprotozoal activity and does not disrupt the protective aerobic microbiota [50, 51]. Combined administration of amoxicillin and metronidazole could cover most of the oral bacteria [43]. Prescription of this combination or metronidazole is also recommended for the treatment of periodontal infections [40, 52, 53]. The drug is commonly prescribed with a dosage of 500–750 mg every 8 hours [21].

The dental practitioners should bear in mind that metronidazole can interact with some agents such as alcohol (causes nausea, vomiting, and abdominal cramp), disulfiram, warfarin, and hydantoin anticonvulsants [50]. The agent might also result in serious side effects, such as seizures, anesthesia, or paresthesia of the limbs in certain patients [21]. Two cases with metronidazole resistance have been reported in Scotland: one was an infection of the knee joint (with anaerobic streptococci that is found in dental abscess and periodontal disease) and the other was *Bacteroides thetaiotaomicron* bloodstream infection [51].

### 5.4. Macrolides

Macrolides have a macrocyclic lactone ring, which are bacteriostatic agents that inhibit protein synthesis; these agents have translation modulators that act against bacterial ribosomes [54–56]. Macrolides mainly act against beta-hemolytic streptococci [57]. Macrolides should not be coadministered with clindamycin, since these have the same target point and antagonistic effects [56]. Moreover, macrolides should not be prescribed in patients with progressive cirrhosis, as this could result in liver failure and even death [42].

#### 5.4.1. Erythromycin

Erythromycin has bacteriostatic activities and is commonly prescribed for dental caries and dental plaque [55, 56, 58]. The most common microorganism that causes dental caries is *Streptococcus mutans*, which is highly sensitive to erythromycin [59]. Erythromycin can inactivate the caries, and it also can decrease the growth and formation of dental plaque [60].

Erythromycin should be prescribed with a dosage of 250–500 mg every 6 hours [13]. However, the drug is not regularly recommended as it could cause several short-term and long-term adverse effects, such as gastrointestinal problems, hepatotoxicity, and also bacterial resistance [61]. Moreover, the drug is contraindicated in patients taking simvastatin or colchicine and also in patients who suffer from porphyria [62].

#### 5.4.2. Azithromycin

Azithromycin is a bacteriostatic antibiotic that has a great potency against Gram-negative pathogens and is considered to be the safest among the macrolides [56, 63]. The drug is not suggested as the first-line treatment of odontogenic infections and is usually prescribed as an alternative in penicillin-allergic patients [63, 64].

The dosage of the drug is 500 mg once a day for three days, in case of therapeutic prescription, and 500 mg 1 hour before the oral procedure, in case of prophylactic administration [13, 46]. The common side effects of azithromycin include nausea, diarrhea, and gastrointestinal disorders, and it should not be prescribed in erythromycin-allergic patients [21, 56, 63].

#### 5.4.3. Clarithromycin

Clarithromycin is a broad-spectrum antibiotic that is considered to be the new generation of erythromycin [65]. Clarithromycin is a bacterial protein synthesis inhibitory and matrix metalloproteinase (MMP) regulating activities that could fight against intracellular pathogens by penetrating the cells [66]. Among the macrolides, the agent is believed to have the greatest effect against anaerobic Gram-positive bacilli [6]. Hence over, the prescription of clarithromycin can be a logical approach for suppressing the pulp and periodontal infections [67, 68]. However, clarithromycin is not usually recommended as the first-line treatment and is used instead of penicillin in patients who cannot tolerate the gold standard treatment of penicillin [62].

The standard dose for prophylaxis is 500 mg orally 1 hour before the dental procedure [46]. The most common side effects of clarithromycin are gastrointestinal complications, such as nausea and diarrhea [61]. It is indicated that clarithromycin has some new effects such as modulating myocarditis, cardiac rejection, and change of inflammatory signs [67].

### 5.5. Lincosamides

Lincosamides are bacteriostatic agents that mostly fight against Gram-positive anaerobic pathogens, by binding to the functional spot of the bacterial ribosome and restricting the protein synthesis [69, 70]. Lincomycin and clindamycin are the drugs that are classified in the group of lincosamides antibiotics [71]. Studies showed that clindamycin has a greater effect against infections compared with other lincomycin [69]. The coadministration of lincomycin and erythromycin is not suggested, as these two drugs have an antagonistic effect against each other [72]. While, among lincosamides, the prescription of clindamycin is more common than the others [70].

#### 5.5.1. Clindamycin

Clindamycin is a broad-spectrum bacteriostatic antibiotic that covers both aerobic and anaerobic pathogens [73, 74]. The drug is the newer generation of lincomycin, and it has suitable potency against bone, joint, and odontogenic infections [73, 74]. As showed by the previous investigations, nearly 75% of all bacteria causing odontogenic infections are sensitive to the drug [33]. Clindamycin could be prescribed in the case of persistent infections, as it has more efficacies in comparison with penicillin and metronidazole [73]. Besides, it has been shown that the bacterial resistance rates against penicillin are higher comparing to clindamycin [75]. Moreover, the agent could be administered IV or IM, besides, oral ingestion [76].

Clindamycin is also an excellent choice for patients who have an allergy to beta-lactam group antibiotics. The therapeutic dosage of the drug is 600 mg or 300 mg every 8 hours orally or intravenously [2, 6, 13]. The drug is also a proffered alternative for prophylaxis in penicillin-allergic patients for prophylaxis [20]. The usual prophylactic dose is 600 mg before procedure orally or 600 mg intravenously in both penicillin-allergic patients and those who cannot take medication by mouth [20]. Furthermore, more recent studies showed that clindamycin might reduce the risk of dry socket after extraction [46].

The most common side effects of clindamycin are vomiting, nausea, diarrhea, exanthem, jaundice, hepatitis, neutrophil reduction, eosinophilia, agranulocytosis, blood platelet count change, and pseudomembranous colitis [68, 70]. The agent is contraindicated for cirrhotic patients and for patients with a history of ulcerative and pseudomembranous colitis [73, 75, 76].

### 5.6. Fluoroquinolones

Fluoroquinolones are broad-spectrum bactericidal antibiotics that mostly act against Gram-negative bacilli, Gram-positive aerobic cocci, and anaerobic organisms, by preventing the synthesis of DNA [77–80]. Fluoroquinolones are commonly prescribed for nonodontogenic infections, such as respiratory, genitourinary tract, joint, and bone infections [78]. These agents have a higher capacity of penetration into tissue in comparison with other commonly prescribed antibiotics in dental practice [81].

The side effects of this class of antibiotics include gastrointestinal reactions and cartilage, joint, tendon, and the central nervous system involvement [82, 83]. Fluoroquinolones must not be prescribed for children because of the possibility of chondrotoxicity in developing cartilage and for patients who use theophylline, as this could result in serious complications, for example, seizure [79].

#### 5.6.1. Ciprofloxacin

Ciprofloxacin is among the second generation of fluoroquinolone antibiotics and is active against Gram-positive and Gram-negative pathogens [53, 77]. This antibiotic showed excellent antibacterial potency, whilst having minimum side effects [56, 84, 85]. The drug is usually administered orally with a dosage of 500 mg every 12 hours to treat odontogenic infections [20]. The most common side effect of ciprofloxacin is gastrointestinal problems, including, nausea, vomiting, and diarrhea [21]. Dental practitioners should take the patients' history as if they have been using theophylline because the drug interaction could result in severe consequences [86]. The initial signs of theophylline toxicity in these patients are nausea and vomiting, which should not be confused with the side effects of ciprofloxacin [86].

#### 5.6.2. Moxifloxacin

Moxifloxacin is a broad-spectrum bactericidal agent and a fourth-generation fluoroquinolone. The drug acts against aerobic, anaerobic, Gram-positive, and Gram-negative bacteria and is commonly administered to control chronic bronchitis, pneumonia, skin infections, and bacterial sinusitis [53, 75]. Prior investigations showed that most of the bacterial populations found in odontogenic infections are susceptible to moxifloxacin [33].

Moxifloxacin can be considered as a good choice to treat odontogenic and periodontal infections as well, since it has high penetration capacity through periodontal and bone tissues [56, 81, 87, 88]. Moreover, this could be prophylactically prescribed to beta-lactam-allergic patients to prevent bacteremia [64]. However, moxifloxacin is not used as the first-line treatment because of its high price and is usually prescribed when the first-line antibiotics and surgical procedures are failed [46, 79]. The effective dose of the agent to control odontogenic infections is 400 mg once a day [79]. The major concern is that the drug could affect cartilage maturation; hence, it must not be in pregnant and adolescent patients [56].

### 5.7. Tetracyclines

Tetracycline is a bacteriostatic antibiotic that is active against Gram-positive and Gram-negative bacteria, acting by blocking the synthesis of protein through binding to the ribosomal subunit [89]. The drug could be a reasonable prescription for the treatment of periodontal diseases, as it has anti-inflammatory activity, collagenase inhibition potential, and bone resorption inhibitory capacity; besides, it could help the fibroblasts to attach to the root surface [90].

Tetracycline is recommended in cases of periodontal diseases, improving marginal attachment and enhancing bone graft [56, 90]. The drug has a long half-life, preserves its antimicrobial activity for a long time, and is released from the tooth surface gradually [90]. However, the agent is not commonly suggested for the treatment of odontogenic infections because of the widespread resistance of pathogens and several side effects, including photosensitivity, nausea, vomiting, diarrhea, loss of appetite, hepatotoxicity, and discoloration of primary and permanent teeth [46, 91]. The prescription of the drug for young children and pregnant women is not recommended because it can cause intrinsic tooth staining during the calcification phase [56, 92]. Besides, tetracycline must not be prescribed for patients with active liver diseases [20].

## 6. Conclusion

Antibiotic therapy is crucial to control dental infections after surgical interventions such as incision, drainage, and pulp debridement. Dentists prefer to prescribe amoxicillin and metronidazole or co-amoxiclav to control dental infections. Moreover, clindamycin is an alternative drug in penicillin-allergic patients. The accurate information about oral microorganisms, the character of oral infections, and the pharmacokinetics of antibiotics reduce the risk of incorrect antibiotic prescription. Some alternative methods exist for treating infection such as low-level laser (LLL) therapy and photodynamic therapy (PDT).

Previous studies show the effectiveness of LLL therapy on infected wounds; moreover, it can reduce inflammation and bacterial proliferation. PDT has been successfully used to eliminate pathogens and treat localized infections such as periodontal infections, abscesses, oral and dental infections, wound, burn, and ear infections. Accurate use of antibiotics is crucial for the treatment of dental infections; accordingly, comprehensive antimicrobial prescribing guidance should be established for dental professionals.

## Figures and Tables

**Table 1:** Different rates of antibiotic prescription by dentists [10].

Antibiotic	Prescription rate (%)
Amoxicillin	51.1
Amoxicillin + clavulanic acid	24
Clindamycin	6.6
Azithromycin	5.3
Clarithromycin	4.4
Doxycycline	3.6
Spiramycin	2.2
Erythromycin	1.2
Ciprofloxacin	0.2
Cefadroxil	0.1
Minocycline	0.1
Cefuroxime	0
Others	1.1

**Table 2:** Therapeutic antibiotic dose for children.

Agent	Situation	Dose	Maximum dose	Available forms
Amoxicillin	First choice in dental infection	20–40 mg/kg/day, e8 h	2 g/day	Tablet 125 mg, capsule 250 mg and 500 mg, and oral suspension 125 mg/5 ml and 250 mg/5 ml
Amoxicillin + clavulanic acid	Failure of first choice antibiotic		1000–2800 mg amoxicillin/143–400 mg clavulanic acid	Tablet 375 mg, 625 mg, and 1000 mg and oral suspension 228.5 mg/5 ml
Clindamycin	Penicillin hypersensitivity	10–20 mg/kg/day, e6 h		Suspension 75 mg/5 ml
Cephalexin	Necessity of broad-spectrum action	25–100 mg/kg/day, e6_8 h		Tablet 125 mg, 250 mg, and 500 mg, capsule 250 mg, 500 mg, and 750 mg, and oral suspension 125 mg/5 ml and 250 mg/5 ml
Metronidazole	Anaerobic bacteria	30 mg/kg/day, 8 h	2 g/day	Tablet 200 mg, 250 mg, 400 mg, and 500 mg, infusion solution 500 mg/5 ml, and oral suspension 200 mg/5 ml

**Table 3:** Antibiotic prophylaxis regimen for children.

Agent	Situation	Administration route	Dosage
Amoxicillin	First choice	Oral	50 mg/kg
Ampicillin or cefazolin/ceftriaxone	Unable to take oral medication	IM or IV	50 mg/kg
Cephalexin	Allergic to penicillin or ampicillin	oral	50 mg/kg
Clindamycin			20 mg/kg
Azithromycin/clarithromycin			15 mg/kg
Cefazolin/ceftriaxone	Allergic to penicillin and ampicillin and unable to take medication orally	IM or IV	50 mg/kg
Clindamycin			20 mg/kg

**Table 4:** FDA risk classes of antibiotics used during pregnancy.

Category risk factor	Antibiotics	Side effects
A		
Satisfactory well-controlled studies on humans showing no hazard to the fetus		
B	Amoxicillin	
Animal studies demonstrate no risk, but no human studies have been performed or human studies have demonstrated no risk	Cephalexin	
	Chlorhexidine	
	Clindamycin	
	Erythromycin	
	Metronidazole	
	Penicillin	
	Azithromycin	
C	Ciprofloxacin	Chondrotoxic in growing rats
Studies on animals establishing fetal hazards and no exact studies on human being	Moxifloxacin	Chondrotoxic in growing rats
	Clarithromycin	Increased risk of miscarriage
D	Doxycycline	Intrinsic dental staining
Evidence of risk to the fetus can be used in exceptional cases or circumstances	Tetracycline	Intrinsic dental staining
X		
The hazards of using the drug in pregnant women far more than the benefits		
